# Effects of Stocking Rate and Environmental Enrichment on the Ontogeny of Pecking Behavior of Laying Hen Pullets Confined in Aviary Compartments during the First 4 Weeks of Life

**DOI:** 10.3390/ani12192639

**Published:** 2022-09-30

**Authors:** Angela Schwarzer, Michael Erhard, Paul Schmidt, Miriam Zismann, Helen Louton

**Affiliations:** 1 Department of Veterinary Sciences, Chair of Animal Welfare, Ethology, Animal Hygiene and Animal Husbandry, Faculty of Veterinary Medicine, LMU Munich, Veterinaerstrasse 13/R, 80539 Munich, Germany; 2 Statistical Consulting for Science and Research, Große Seestraße 8, 13086 Berlin, Germany; 3 Animal Health and Animal Welfare, Faculty of Agricultural and Environmental Sciences, University of Rostock, Justus-von-Liebig-Weg 6b, 18059 Rostock, Germany

**Keywords:** pullet, rearing period, feather pecking, behavior, environmental enrichment, stocking rate

## Abstract

**Simple Summary:**

The objective of this study was to describe the development of aggressive pecking, gentle feather pecking and severe feather pecking of non-beak-trimmed laying hen pullets during the first 4 weeks of life. The pecking behavior patterns were observed in three different experimental groups, which differed in stocking rates and the provision of enrichment material. The enrichment materials were suitable and intensely used by the pullets. The provision of pecking blocks and pecking stones is recommended as a preventive measure from the first day of life onwards for pullets housed in commercial rearing aviaries. There was no effect of reduced stocking rate, most likely due to the low variation in stocking rates.

**Abstract:**

The objective of this study was to describe the ontogeny of the severe feather pecking (SFP), gentle feather pecking (GFP), aggressive pecking (AP), and enrichment pecking (EP) of non-beak-trimmed Lohmann Brown (LB)-pullets during the first 4 weeks of life (observation on 1st, 8th, 15th, 22nd, and 29th days of life) while they were kept within the compartments of a commercial rearing aviary (without access to a litter area). All chicks were placed into compartments of the middle tier of the aviary on the 1st day of life. On the 10th day of life, half of the chicks of each compartment were moved into the compartments of the lower tier. The aviary floor was covered with chick paper before the placement of the chicks and fully or partially removed from the 15th day of life onwards. The pecking behavior patterns were observed in three experimental groups (EG): NE (not enriched): group size until/after 10th day of life: 230/115; 120.8/60.4 birds/m^2^, no enrichment; EL (enriched, low stocking rate): group size until/after 10th day of life: 203/101, 106.6/53.6 birds/m^2^; and EH (enriched, high stocking rate): group size until/after 10th day of life: 230/115;120.8/60.4 birds/m^2^, both pecking stones and blocks as enrichment) in two rearing periods. For each pecking behavior pattern, an independent regression model with the parameters EG, chick paper, observation day, and functional area was estimated. GFP, SFP, and EP increased with age during the observation. The AP rate was highest in all EGs on the first day of life and decreased during the observation period. A pairwise comparison of NE (high stocking rate without enrichment) with EH (high stocking rate with enrichment and with EL (low stocking rate with enrichment) showed a significant effect of the EG on pecking behavior, with more SFP, AP, and GFP in NE. There were no differences between EL and EH, indicating that the provision of pecking materials had more influence than the stocking rate. However, we presumed that the difference between the stocking rates were too small to observe an effect. AP, SFP, and GFP were significantly higher on wired slats, as compared to the perches and the vicinity of the enrichment materials. The enrichment materials were suitable and intensely used by the pullets. The provision of pecking blocks and pecking stones was recommended as a preventive measure from the first day of life onwards for pullets housed in commercial rearing aviaries. There was no effect of reduced stocking rate, most likely due to the low variation in stocking rates.

## 1. Introduction

Severe feather pecking (SFP) is a behavioral disorder of layers [1] that constitutes both a severe animal welfare problem, due to pain [2], and an economic problem [3]. SFP is not motivated by aggression [4,5,6,7]; however, another study found a positive correlation between SFP and aggressive pecking (AP) [8]. Foraging behavior, including ground pecking, is congenital [9]. Gentle feather pecking (GFP) is defined as the soft pecking against the plumage of another pullet without plucking or pulling feathers [4]. It is part of normal social exploration behavior, with low frequency and intensity [4,10]. GFP was observed from the first week of life onwards [10] with high frequency during the first six weeks of life [11]. GFP was more frequent after housing unfamiliar birds together [12]. GFP might develop into stereotypic pecking [13] and consequently lead to SFP; however, the influencing factors for this development are not yet known [12]. AP is part of normal behavior to establish a pecking hierarchy to defend resources such as food [4,5,14]. Significantly more AP was found in laying hen flocks without males [4,7] and after being housed with unfamiliar hens [5]. AP has been described in pullets from the 10th day of life [9]. Laying hens can establish pecking orders up to approximately 100 birds [14,15]. Individual recognition and establishing pecking orders is not possible in large flocks (>60 birds) [16], and it is likely that the social organization changes in larger flocks. Aggression decreases, and the social organization is based on their tolerance for other birds, without individual recognition [17,18].

In multi-tier commercial rearing aviaries, pullets are kept inside separated compartments of the aviary for approximately the first 4–5 weeks of life [19,20]. According to Gilani et al. [21], both GFP and SFP may develop during rearing on commercial farms. The rearing conditions are an important risk factor for the development of SFP in laying hens [22,23,24,25,26]. There is some evidence that the stressful experience in the commercial hatchery has an overall negative effect on the development of pullets [27]. The combination of not having litter at the age of 1–4 weeks and the absence of daylight at the age of 7–17 weeks was a significant predictor of feather damage during the laying period in organic laying hens according to Bestman et al. [23]. Similar results were found in an experimental study, which compared rearing on littered floors with wired floors [28] and for commercial rearing farms [29]. A field study in the United Kingdom with 34 flocks found good foraging behavior to be one of the most important factors in preventing feather pecking [21]. The importance of early access to litter and reduced stocking rates for the prevention of feather pecking has been reviewed in Nicol et al. [30].

Low stocking rates have had a positive effect on reducing SFP during the rearing period [31,32,33,34,35,36]. Other authors, however, did not confirm this relation [26,37]. High stocking rates had a negative influence on foraging behavior due to a lack of space [38].

Recommendations for stocking rates to minimize the risk of SFP and cannibalism range from 10 pullets/m^2^ to a maximum of 18 pullets/m^2^ [19,32,36]. A calculation considering the size of the pullets and the relative additional space yielded between 9–15 pullets/m^2^ [38]. While confined in the aviary, a maximum of 100 pullets/m^2^ up to the 10th day of life and 50 pullets/m^2^ until the pullets get access to the entire aviary, including a litter area, has been recommended [19]. Pullets housed in overcrowded conditions (179/90 chicks/m^2^ 0–3 and 3–6 weeks of age, respectively) during the first six weeks of life were found to have more anxious behavior, compared to pullets housed in undercrowded conditions (20/10 pullets/m^2^ 0–3 and 3–6 weeks of age, respectively), and both groups had higher corticosterone levels, as compared to pullets housed in conventional crowding (60/30 pullets/m^2^ 0–3 and 3–6 weeks of age, respectively). The authors concluded that conventional stocking rates did not impair the welfare state of pullets but emphasized that an increase in the stocking rate led to a slower rate of adaptation, implicating long-term consequences from the different stocking rates and/or increased costs of adaptation [39]. Lower stocking densities along with reduced flock sizes also encouraged pullets to use a range from 8 weeks of life onwards [40]. The percentage of birds seen using the range was higher with reduced flock size and stocking density, increased pop hole availability (cm/bird) and light intensity inside the house.

Regarding the location of feather pecking in the housing system, the studies of Johnsen et al., and of Hansen, observed most feather pecking bouts on the perches, followed by the litter area and feeding platforms [22,31]. Other authors reported feather pecking predominantly in the litter area [24,41,42] or on the aviary platforms [43]. A layer of chick paper with food placed on top is usually put on the aviary slats before placing the pullets as part of the coccidiosis vaccination and feed-management strategy [20]. However, the chick paper usually fades away within 10–14 days, and after that, the pullets stay on wired slats until given access to the litter area [19]. Tahamtani et al. found that pullets reared on chick paper from the first day of life had less plumage damage by the 30th week of life [44]. There was a significant decrease in foraging behavior after the removal of the chick paper [44]. Environmental enrichment could reduce feather pecking during rearing [26] and in the laying period [45]. Abrasive pecking pans were better accepted by 6-week-old pullets, compared to pullets at the end of the rearing period, and effectively blunted the beaks of non-beak-trimmed birds [46]. However, another study found that the provision of novel objects during rearing had only minimal effects on pullet behavior and welfare [47].

The objective of this study was to describe the ontogeny of the pecking behavior of laying hen pullets under conventional housing conditions during the first four weeks of life while confined within an aviary compartment (without access to a litter area). SFP, GFP, and AP, as well as pecking at the enrichment materials (EP), were observed in flocks with different stocking rates, with and without the provision of environmental enrichment. The unique aspect of this paper was the analysis of the different pecking behavior patterns (EP, GFP, SFP, and AP) of laying hen pullets in a conventional rearing aviary before the pullets had access to the litter area and the remaining tiers of the aviary.

## 2. Materials and Methods

### 2.1. Farm, Animals, and Management

The study was conducted in a conventional rearing farm in Bavaria, Germany. We observed non-beak-trimmed Lohmann Brown Classic (LB)-pullets during two consecutive rearing periods (RP). Feeding and management were the same for all animals according to the guidelines of the breeding company [20]. All pullets were vaccinated according to the scheme recommended by the breeding company [20].

The rearing aviary (Type 501-3, Meller Anlagenbau GmbH, Melle, Germany) had 3 tiers, and each tier was divided into several compartments (80 cm × 240 cm each). Each compartment was equipped with 8 nipple drinkers, a feeding chain, and a perch above the feeding chain and the drinking line (240 cm length each). The floor of the compartments consisted of wire slats (17 cm × 36 mm) with a manure belt underneath each slat to prevent manure from falling on animals housed on the tier underneath. In both RP1 and RP2 the slats were covered with chick paper before the placement of the chicks, so that chick paper was available for all chicks during the observation days of life 1 and 8. Chick paper is used to cover the wired slats of rearing aviaries and to spread feed on the surface before placing the chicks to facilitate feed intake [20]. It was partially or completely removed on day 15, according to the standard management procedures of the farm. During the rearing period the remains of the chick paper disappeared gradually, so that no chick paper was left in the aviary compartments at the end of the observation period. In all compartments high frequency neo tubes were used for lighting, there was no daylight. Results for the analysis of the light on animal health are published in Liebers et al. [48]. All chicks were placed into the compartments of the middle tier of the aviary on the 1st day of life. The pullets of the RP1 were placed in the rearing barn on 13 June 2015, the chicks of RP2 on 15 December 2015. After ten days, half of the chicks of each compartment were moved into the compartments of the lower tier of the aviary due to their increasing space requirements. On day 32 of life, the aviary was opened, and the pullets gained access to the entire aviary, including the litter area on the ground. Zepp et al. presented the results of the pecking behavior after the pullets had access to the entire aviary, including the litter area [35].

### 2.2. Study Design

We created 3 experimental groups (EG) that differed in stocking rate and the provision of environmental enrichment: NE (not enriched), EL (enriched, low stocking rate) and EH (enriched, high stocking rate, Table 1). Each of the 3 EGs was observed in 8 compartments in total (4 compartments per EG in RP1 and in RP2). We used pecking stones (VILOLith^®^PICKStein Geflügel, Fa. Vilomix, Neuenkirchen-Vörden, Germany) and pecking blocks (PICKBLOCK™, Fa. Crystalyx, Münster, Germany), which had feed approval as environmental enrichment materials (EM) for EL and EH. The mineral-based pecking stones were round and weighed 8 kg. The pecking blocks were rectangular, weighed 5 kg each, and consisted of a mixture of organic grain, minerals, and fiber. Each compartment of EL and EH was equipped with one-sixth of a pecking stone and one-quarter (RP1) or one-half (RP2) of a pecking block. They were not replaced until the end of the observation period (day 29 of life). As the pecking block was almost entirely consumed by the end of the aviary confinement period (day 32 of life) in RP 1, we used ½ pecking block instead of ¼ pecking block in RP2. A reduction in the usable area by the pecking materials was not taken into account, as the chicks could still hop and sit on them, and the size of the pecking block decreased constantly due to consumption. Data regarding animal health, microclimate, and the use of environmental enrichment in all experimental groups were published in Liebers et al. [45].

### 2.3. Video Observation

We used 24 VTC-E220IRP SANTEC color cameras with infrared-light emitting diodes (Santec BW AG, Ahrensburg, Germany). For recording and saving the video recordings, the Indigo Vision (Indigo Vision Ltd., Edinburgh, UK, GB) software was used.

We installed two cameras per compartment in the upper left and right corners to cover almost the entire compartment, including the EM. The pecking behavior was recorded according to the definitions described in Table 2. The different pecking behavior patterns of the pullets (Table 2) were analyzed on the days of life 1 (initial placement), 8, 15, 22, and 29, using the “continuous recording” method [49]. The 3 min continuous recording intervals were conducted 5 times per day on days of life 1 and 8 (intermittent light phase: 4 light phases of 2–6 h/24 h) and 3 times per day on days of life 15, 22, and 29 (continuous light phase, 16 h light on days 15 and 22, 16 h light on day 29). On days of life 1 and 8 we analyzed 15 min and on the remaining days (15, 22 and 29) 9 min per day and compartment. On day 1 and 8, continuous recording took place 1 h after the light was switched on plus a second time 1 h before the light was turned off in the 6-h-light phase in the afternoon. On days of life 15, 22 and 29, continuous recording was conducted 1 h after the light was switched on and off, respectively, and additionally in the middle of the light day after 8 h. Each continuous recording interval was 3 min long. We recorded in which part of the aviary section the pecking behavior was observed. For this purpose, we classified 3 functional areas within the compartment: “wired slat” (both in front of and behind the feeding chain), “enrichment” (on top of or right next to a pecking block or stone, EL and EH only), and “perch” (above the feeding chain and above the nipple drinkers). The video analysis was conducted by one researcher only, who performed several training sessions before data recording.

### 2.4. Statistics

All data were processed using Microsoft Excel 2016 (Microsoft Corporation, Redmond, WA, USA). Each compartment was considered one statistical unit, resulting in *n* = 8 per EG. For further statistical analysis, the programming language R was used [50]. The total number of pecks of each pecking behavior (SFP, GFP, AP and EP) in each 3 min continuous recording session was divided by the number of birds visible on the camera in that session, resulting in the value of pecks per bird per three-minute period (pecks/bird/3 min) to account for different stocking rates. After descriptive analysis, independent linear mixed regression models were estimated for each pecking behavior. In these models, pecking behavior was used as the dependent variable and the parameters experimental group (EG), chick paper, observation day, rearing period, and functional area were included as fixed effects and the compartment as a random effect for the intercept. Evolution of the pecking behavior over time was estimated along rearing periods by including the interaction between the observation day and rearing period in the models. The same procedure was applied to the effect of the functional area. In a similar way, the effect of chick paper was estimated along with the observation day. Here, the models were extended by the interaction between chick paper and observation day.

The correlation between enrichment pecking and the consumption of the enrichment materials was analyzed using Pearson’s correlation coefficient and linear mixed models with consumption of the enrichment materials as the dependent variable, enrichment pecking, observation day and rearing period as fixed effects, and the compartment as a random effect for the intercept. In order to assess the consumption effect along with the rearing period, the model was extended by the corresponding interaction. The same approach was applied to the effect of consumption along with the observation day.

## 3. Results

### 3.1. Ontogeny of the Pecking Behavior

**Figure 1:**
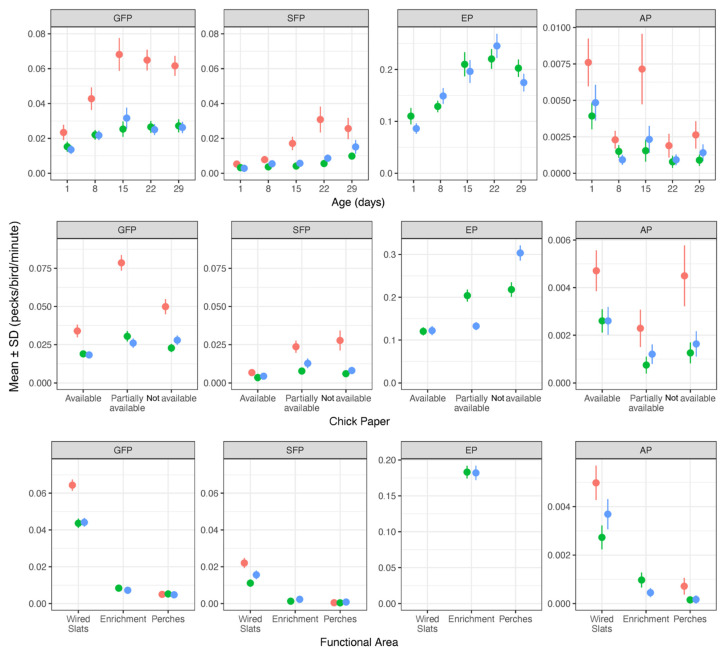
Descriptive analysis (mean +/– SD pecking pecks/bird/min) of the occurrence of gentle feather pecking (GFP), severe feather pecking (SFP), enrichment pecking (EP), and aggressive pecking (AP) in the experimental groups. Red = NE (not enriched, high stocking rate), green = EL (enriched, low stocking rate), blue = HE (enriched, high stocking rate). Upper row: pecking behavior on the five observation days (days of life 1, 8, 15 22, and 29), middle row: pecking behavior depending on the availability of chick paper, lower row: pecking behavior in the different functional areas. Behavior was observed in eight compartments per experimental group (NE, EL and EH) in both rearing periods with two cameras per compartment. On days of life 1 and 8 we analyzed 15 min and on the remaining days (15, 22 and 29) 9 min per day and camera.

We observed 2147 pecks of GFP and 635 pecks of SFP, in total, starting on the first day of life. The overall descriptive analysis showed an increase in SFP, GFP, and EP (excluding the last observation day) and a decrease in AP with age for all experimental groups. Both GFP and SFP occurred at very low rates on the 1st day of life, but rates increased steadily with age. However, the pullets in NE had higher pecking rates (GFP, SFP, and AP) than EL and EH (Figure 1). NE could not perform EP, as this group did not have access to pecking stones or pecking blocks. Aggressive pecking occurred the least often, compared to feather and enrichment pecking (184 pecks in total), and was observed most often on the first day of life in all EGs. NE had another peak in AP on the 15th day of life, while the AP rates for EL and EH remained low for the remaining observation days (Figure 1). There was no significant effect of the time of day (morning and afternoon) on the occurrence of SFP, GFP, AP and EP, both over all five observation days and separately for the five observation days. The regression model showed significant differences between observation days for NE regarding the occurrence of SFP and GFP. As compared to day 1, pullets in NE had a significantly higher GFP rate on days of life 8 (95% CI 0.025–0.089, *p* < 0.001), 15 (95% CI 0.029–0.107, *p* < 0.001), 22 (95% CI 0.018–0.09, *p* = 0.003), and 29 (95% CI 0.012–0.081, *p* = 0.009). The SPF rate was significant on days of life 22 (95% CI 0.032–0.086, *p* < 0.001) and 29 (95% CI 0.017–0.07, *p* < 0.001), compared to day of life 1, and on days of life 22 (95% CI: 0.025–0.078, *p* < 0.001) and 29 (95% CI: 0.01–0.062, *p* = 0.006), compared to days of life 8, and finally between days of life 15 and 22 (95% CI: 0.013–0.072, *p* < 0.004). Comparable effects on the observation days were not estimated for the two experimental groups (EL and EH) that had access to enrichment materials. For AP, significant differences were observed between most days of life for NE and between some days of life for EL and EH. In most cases, these differences were more pronounced for NE.

### 3.2. Effect of the Experimental Group on Pecking Behavior

**Figure 2:**
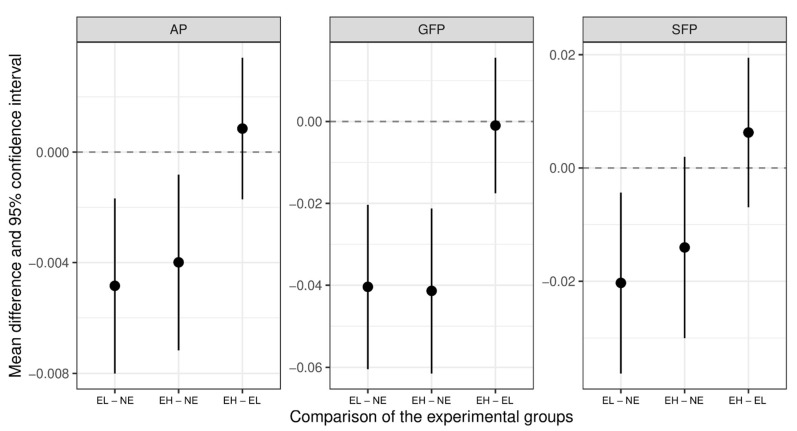
Estimated effects of the experimental group on the occurrence of pecking behavior. The diagram shows the estimated effect and 95% confidence intervals (CI) of the experimental group (EG) on the occurrence of gentle feather pecking (GFP), severe feather pecking (SFP), and aggressive pecking (AP). Results were considered significant when the CI did not include zero. Estimated effects above zero indicate an increase in the chance of observing a behavioral parameter, whereas estimated effects below zero indicate a decrease. Experimental groups: NE (not enriched, high stocking rate), EL (enriched, low stocking rate), EH (enriched, high stocking rate).

A pairwise comparison revealed significantly higher rates of all pecking behaviors for NE, compared to EL and EH (Figure 2), except for NE vs. EH for SFP. EL and EH showed significantly lower rates of AP (CI for the difference between NE and EL −0.008–−0.002, CI for the difference between NE and EH −0.007–−0.001), SFP (CI for the difference between NE and EL −0.036–−0.004, CI for the difference between NE and EH −0.030–0.002), and GFP (CI for the difference between NE and EL −0.060–−0.020, CI for the difference between NE and EH −0.062–−0.021), compared to NE.

### 3.3. Pecking Behavior in Different Functional Areas

**Figure 3:**
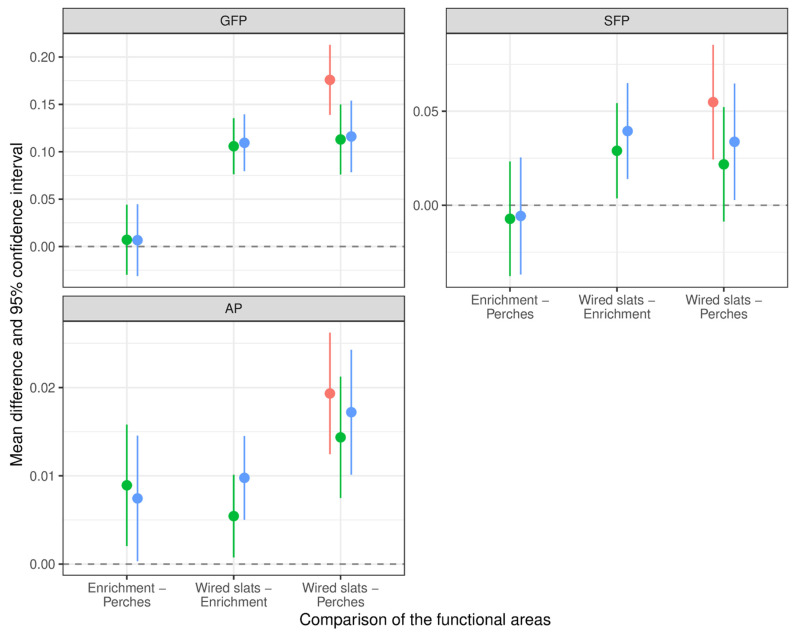
Mean differences of functional areas on the occurrence of pecking. The diagram shows the mean differences and the 95% confidence interval (CI) of observed functional areas on the occurrence of gentle feather pecking (GFP), severe feather pecking (SFP), and aggressive pecking (AP). Results were considered significant when the CI did not include zero. Estimated effects above zero indicate an increase in the chance of observing a behavioral parameter, whereas estimated effects below zero indicate a decrease. Red = NE (not enriched, high stocking rate), green = EL (enriched, low stocking rate), blue = EH (enriched, high stocking rate).

The descriptive analysis showed that SFP, GFP, and AP occurred predominantly on the wired slats in all EGs (Figure 1). Pecks on the perch or close to the enrichment materials were rarely observed. EP could only be observed next to the pecking blocks and stones (Table 3). The regression model revealed that both GFP (95% CI 0.093–0.136) and SFP (95% CI 0.020–0.055) were observed significantly more often on the wired slats, compared to the area close to the enrichment materials and the perches (Figure 3). The difference in the occurrence of GFP between the wired slats and the perches was significantly higher for NE compared to EL (*p* = 0.003) and EG3 (*p* = 0.006). For SFP, a difference between NE and EL cannot be confirmed. For AP, there was the same tendency, although it failed to reach significance.

### 3.4. The Influence of Chick Paper on Pecking Behavior

All chicks had chick paper available at the beginning of the rearing period (until the 8th day of life). A descriptive analysis of the effect of chick paper on the different pecking behaviors showed higher rates of EP, SFP, and GFP but lower rates of AP if the chick paper was only in parts of the compartment or was not available on observation days of life 15, 22 and 29 (Table 3). Chick paper influenced enrichment pecking and aggressive pecking. There was significantly more EP on the 22nd day of life if the chick paper was not available, (95% CI 0.3753–0.7761) as compared to being partially available (covering parts of the wire floor of the compartment). On the 15th day of life, we found significantly more AP if there was no chick paper available (95% CI 0.0064–0.0176). For SFP, there were no significant effects.

### 3.5. Consumption of Enrichment Materials

Over all five observations days (days of life 1, 8, 15, 22, and 29), there was a positive correlation between the amount of pecking stones consumed per day (g) and the EP rate (EL: *r* = 0.431; EG3: *r* = 0.437). Further analysis showed a significantly higher consumption of the pecking stones with rising pecking rates (95% CI 0.25–0.42). The positive correlation between EP and the consumption of the pecking material was significant for the observations from days of life 22 (95% CI 0.081–0.939) and 29 (95% CI 0.121–0.819). There were no differences between the two groups.

## 4. Discussion

In this study all forms of pecking behavior (SFP, GFP, AP, and EP) were seen from one day old, whereas other studies have observed AP from the 2nd week of life onwards only [9,14], except EP, which could not be performed in NE due to a lack of provision of Enrichment. The overall pecking rate increased with age and distinguished the group differences. While SFP, GFP, and EP rates were very low on day of life 1 and increased with age, the AP rate was the highest on day of life 1. The pairwise comparison of the effect of the day of life on the pecking rate revealed the most pronounced differences for NE for GFP, SFP, and AP. AP is used to establish a pecking order [14,15]. The possibly of stressful circumstances in a commercial hatchery and housing conditions [26,27] could have facilitated agonistic behavior towards their conspecifics, despite the availability of enough food, which had been spread generously on the chick paper throughout the compartment before the placement of the chicks [20]. However, the establishment of a pecking order was probably not possible due to the large group size, consisting of over 100 animals, which was too large to enable the individual acquaintanceship of the chicks [15,16]. On the following observation days (days of life 8, 15, 22, and 29), the AP rate decreased and remained low, which could have been a sign that the social organization had changed towards a tolerance model, as described by Pagel and Dawkins [17], as well as Estevez et al. [18]. However, the AP rate was always highest in EG1 with a high stocking rate and no enrichment. Only in NE did we observe a peak of the AP rate on the 15th day of life. We presumed this could have been due to stress in the group after placing half of the original group into another compartment on the tenth/eleventh day of life. Furthermore, it was possible that the remaining chicks had not yet settled into the new situation by the 15th day of life, as afterwards, the AP rate decreased again. Eugen et al. described that overcrowded housing might lead to behavioral consequences or difficulties in adaption [39]. EG1 had a higher stocking rate than normal crowding, but not as high as the overcrowded flocks in the study of Eugen et al. [39]. It was possible that the lower stocking rates and/or the provision of enrichment in the other two EGs had prevented this peak in AP after splitting up the group. The behavioral and physiological consequences of this routine management procedure should be investigated in more detail in future research.

There was no difference in the EP rate of EL with a low stocking rate or in EH with a high stocking rate (enrichment being the same in both groups). Starting at a low level, EP rates increased during the rearing period (8th, 15th, and 22nd day of life) but decreased on the 29th day of life. This was attributed to the enrichment materials being nearly expended without replacement at the end of the observation period, which was shortly before the aviary compartments had been opened to give access to the litter area. The provision of half a pecking block in RP2 instead of only a quarter in RP1 might have influenced the EP between the two RPs, but we still saw a decrease in EP at the last observation day (29th day of life) in RP2. Results for pecking behavior in this phase were published in Zepp et al. [35]. All pecking behavior patterns towards other pullets (AP, GFP, and SFP) were predominantly observed while the animals were on the wired slats of the compartment, as compared to the vicinity of the enrichment and the perches. This effect was significant for all EGs; however, it was most pronounced for NE. Other studies found the highest pecking rates in the litter area, as compared to perches and the nest box area in commercial laying hen flocks but did not observe aviary platforms [24,41,42]. The study of Elger, however, found a significant effect of the functional area “aviary platforms” on SFP in laying hens housed in commercial aviaries, compared to the functional areas “litter area” and “perches” [43]. Evidence that SFP was mainly observed on perches [22,31] was not confirmed in this study. On the contrary, there was significantly less GFP, SFP, and AP on the perches compared to the wired slats in all EGs.

Possible influencing factors on the pecking behavior are discussed in the following paragraphs.

Remarkably, both GFP and SFP were predominantly observed in NE, the group with a high stocking rate without enrichment. The pairwise comparison of the groups revealed significant differences: NE showed significantly more SFP, AP, and GFP, compared to EL and EH. Significant differences between pecking behavior on the different days of life between EL and EH, which differed in stocking rate only, were observed only on the 29th day of life, as compared to days of life 1, 8, and 15. Therefore, we speculated that it was likely that the enrichment had more influence than stocking rate. This did not agree, however, with other studies that found an effect from the stocking rate [32,38]. The availability of enrichment did not seem to trigger aggressive pecking in the sense of a competition over this resource and might have had a positive effect on SFP and GFP. However, our results seem to indicate that the presence of enrichment material seem to reduce feather pecking. Enrichment material does indeed seem to distract the birds from SFP and AP, which can lead to injuries. The stocking rates in our study were higher than those in most other experimental studies (similar to stocking rates found in practice), and the variations in stocking rates (NE 120.8/60.4 pullets per square meter before/after the 11th day of life vs. EL and EH 106.6/53.6 pullets per square meter before/after the 11th day of life) was low. Even the low stocking rate in EL was higher than the stocking rates used in previous studies [32,38] and exceeded current recommendations [19,51], due to our aim to conduct this research project under conventional farming conditions found in practice. However, it was 20% lower than the usual stocking rate found in practice that was used for NE and EH. There has been some evidence that such high stocking rates could be considered “overcrowding”, with negative effects on the behavior and adaptation of pullets to their environment [39]. Further studies should investigate whether placing chicks in compartments in both tiers from the first day of life and thus avoiding the splitting of the group, while at the same time reducing stocking rates from days of life 1–10, would have a positive effect on the behavior of the pullets.

Regarding the experimental design there is an unavoidable relation between stocking rate and group size. We used compartments of the commercial aviary and stocking rates could only be reduced by reducing the group size. The differences due to stocking rates might therefore be confounded by different group sizes. It would be interesting for further experimental studies to use constant group sizes with different compartment sizes. As the rearing flocks in this study did not have access to a free range, we could not study whether the reduced stocking rate had an influence on ranging behavior as described by Gilani et al. [40].

As described in the literature [26,45,46], the access to enrichment materials such as pecking stones and pecking blocks in this study appeared to have a positive influence on the reduction in SFP and GFP and did not have adverse effects on animal health [47], which goes in line with the results of our study. Therefore, the use of pecking materials may be recommended from the 1st day of life onwards while the pullets are confined within the rearing aviary. Both pecking blocks and chick paper are presumed to be valuable enrichments to limit the development of SFP. Chick paper is routinely used in practice to cover the wired slats of rearing aviaries and to spread feed on the surface before placing the chicks to facilitate feed intake [20]. During the first two weeks, the chick paper fades and remnants are removed [20]. We investigated a possible effect of the availability of chick paper on the different pecking behavior patterns. We assumed that chick paper with feed and feces crumbles on it might have encouraged the chicks’ foraging behavior, which is preventative for SFP according to Gilani et al. [21]. EP was observed significantly more if chick paper was not available, compared to when chick paper was partially available. However, there was no effect on SFP. In contrast to Iffland et al. [8], who found a positive correlation between AP and SFP, we found an effect on AP only on the 15th day of life. Significantly more AP occurred if the chick paper was not available compared to being partially available. However, less GFP was observed, if there was no chick paper comparted to partial available chick paper. This could have been due to the different underlying motivation for this behavior pattern. GFP is described as part of normal social behavior [4,10], thus an influence of an enrichment material facilitating foraging behavior on GFP is less likely. According to Tahatami et al. [44] chick paper appears to have a positive influence on the behavior. It could, therefore, be useful to keep at least a part of the compartment covered with chick paper until the pullets gain access to the entire aviary, including the litter area. The possible negative hygienic aspects should be addressed in future research.

Additionally, a reduction in the stocking rate to currently recommended values [51] might further reduce feather pecking, even if manipulable environmental enrichment was already available. A range of studies have confirmed the positive effect of low stocking rates on plumage conditions and a lower feather-pecking rate [23,31,32,33,52].

The research results suggested that the availability of environmental enrichment and foraging material had a positive influence on the reduction in feather pecking [26,45,53,54]. Not surprisingly, we found positive correlations between the EP rate and the usage or consumption of the pecking stones and pecking blocks both in EL and EH. This correlation reached significance towards the end of the observation period (days of life 22 and 29), likely due to the chicks growing older and stronger. This result confirms that both enrichment materials used in this study were suitable for and well accepted by the chicks, such as in the study of Baker et al. [46]. As the pecking block was almost entirely consumed by the end of the aviary confinement period (day of life 32) in the 1st RP, we used half a block instead of quarter of a block in RP2. This amount, together with 1/6 of a pecking stone, seemed to be an appropriate amount of pecking material. However, the pecking rate decreased from the 22nd to the 29th day of life. This might indicate that either not enough pecking material was left, that the pecking materials were not accessible to all chicks due to the increasing density in the compartment, or that the pullets had lost interest in the pecking objects. Further research will be necessary to quantify the optimal amount of enrichment materials. As no access to litter during a couple of weeks is a predictor of plumage damage, both during rearing [29,30] and during the laying period on organic farms [23,30], as well as in an experimental study [28], the provision of both litter and additional manipulable enrichment such as pecking objects and chick paper is recommended. Additionally, the abrasive effect of hard pecking objects, such as stones and blocks, can reduce the beak length in pullets [46].

## 5. Conclusions

SFP, GFP, AP, and EP were observed from the first day of life onwards in non-beak-trimmed Lohmann Brown (LB)-pullets in a rearing aviary on a commercial farm. The AP rate was highest in all EGs on the first day of life and decreased during the observation period, with another peak reached in NE on the 15th day of life, indicating stress after the splitting of the group on the 10th/11th day of life. The pairwise comparison of NE (high stocking rate without enrichment) with EH (high stocking rate with enrichment) and EL (low stocking rate with enrichment) showed a significant effect on the pecking behavior, with higher incidences of SFP, AP, and GFP in NE. There were no differences between EL (low stocking rate with enrichment) and EH (high stocking rate with enrichment), "indicating that the provision of pecking materials had a favorable influence on reduction in harmful pecking behavior. Stocking rate did not seem to have an influence on pecking behavior. However, we speculated that the stocking rates in all three EGs were too high overall to observe an effect (120.8 and 60.4 pullets/square meter usable area before/after the 11th day of life). If chick paper partially covered the floor of the compartment, significantly less EP was observed, indicating that chick paper could have served as enrichment material, which encouraged foraging. The enrichment materials were suitable and were intensively used by the pullets. The provision of pecking blocks and pecking stones can be recommended from the first day of life onwards for pullets housed in commercial rearing aviaries to prevent feather pecking. There was no effect from reduced stocking rate, most likely due to the overall stocking rates being too high and the low variance in the experimental groups.

## Figures and Tables

**Table 1:** Overview of the experimental groups NE (not enriched), EL (enriched, low stocking rate) and EH (enriched, high stocking rate): Stocking rates, enrichment, and group sizes. Half of each EG was placed in another compartment of the aviary on the 10th day of life, hence the reduced stocking rates from the 11th day of life onwards. Enrichment: Pecking stones and pecking blocks.

	NE	EL	EH
Group size until 10th day of life (no of pullets)	230	203	230
Group size from 11th day of life (no of pullets)	115	101/102	115
Pullets/m^2^ usable area until 10th day of life	120.8	106.6	120.8
Pullets/m^2^ usable area from 11th day of life	60.4	53.6	60.4
Enrichment (pecking stones and blocks)	No	Yes	Yes

**Table 2:** Definitions of the pecking behavior used for video analysis.

Behavior	Definition
Enrichment pecking (EP)	Pecks directed against pecking blocks or pecking stones
Gentle feather pecking (GFP)	Gentle pecking against the plumage of other birds, without pulling or removing feathers
Severe feather pecking (SFP)	Forceful pecking at or plucking out feathers of other birds
Aggressive pecking (AP)	Forceful, upward–downward pecking directed against the head of another bird

**Table 3:** Descriptive analysis of the average number of pecks (Mean ± SD) depending on the availability of chick paper and in the different functional areas during all observations (days of life 1, 8, 15, 22, and 29) and experimental groups in both rearing periods: Enrichment pecking (EP); gentle feather pecking (GFP); severe feather pecking (SFP); and aggressive pecking (AP). Chick paper was available for all chicks on days of life 1 and 8, and not or partially available on days of life 15, 22 and 29. Behavior was observed in eight compartments per experimental group (not enriched, high stocking rate; enriched, low stocking rate and enriched, high stocking rate) in both rearing periods with two cameras per compartment. On days of life 1 and 8 we analyzed 15 min and on the remaining days (15, 22 and 29) 9 min per day and camera.

	EP	GFP	SFP	AP
Chick Paper	Mean ± SD	Mean ± SD	Mean ± SD	Mean ± SD
Available	0.3633 ± 0.1974	0.0675 ± 0.0848	0.0138 ± 0.0240	0.0094 ± 0.0201
Partially available	0.4980 ± 0.2688	0.1160 ± 0.1184	0.0392 ± 0.0785	0.0038 ± 0.0130
not available	0.7686 ± 0. 3770	0.0910 ± 0.0960	0.0338 ± 0.0772	0.0062 ± 0.0183
**Functional Area**				
Wired Slats	n.a. ^1^	0.1522 ± 0.1021	0.0487 ± 0.0806	0.0114 ± 0.0226
Enrichment	0.5478 ± 0.3361	0.0234 ± 0.0349	0.0053 ± 0.0163	0.0021 ± 0.0092
Perches	n.a. ^1^	0.0150 ± 0.0241	0.0017 ± 0.0063	0.0010 ± 0.0048

^1^ n.a. = not available.

## Data Availability

Not applicable.
